# In vivo safety and biodistribution profile of Klotho-enhanced human urine-derived stem cells for clinical application

**DOI:** 10.1186/s13287-023-03595-y

**Published:** 2023-12-10

**Authors:** Sang-Heon Kim, Sung-Hoon Lee, Jeong-Ah Jin, Hyung-Joon So, Jae-Ung Lee, Min-Jae Ji, Eun-Joong Kwon, Pyo-Sung Han, Hong-Ki Lee, Tae-Wook Kang

**Affiliations:** 1 Institute of Cell Biology and Regenerative Medicine, EHLBio Co., Ltd., Uiwang-si, 16006 Republic of Korea; 2 EHLCell Clinic, Seoul, 06029 Republic of Korea

**Keywords:** Urine-derived stem cells (UDSCs), Safety, Toxicity, Distribution, Tumorigenicity, Klotho, GMP

## Abstract

**Background:**

Urine-derived stem cells (UDSCs) can be easily isolated from urine and possess excellent stem cell characteristics, making them a promising source for cell therapeutics. Due to their kidney origin specificity, UDSCs are considered a superior therapeutic alternative for kidney diseases compared to other stem cells. To enhance the therapeutic potential of UDSCs, we developed a culture method that effectively boosts the expression of Klotho, a kidney-protective therapeutic factor. We also optimized the Good Manufacturing Practice (GMP) system to ensure stable and large-scale production of clinical-grade UDSCs from patient urine. In this study, we evaluated the in vivo safety and distribution of Klotho-enhanced UDSCs after intravenous administration in accordance with Good Laboratory Practice (GLP) regulations.

**Methods:**

Mortality and general symptoms were continuously monitored throughout the entire examination period. We evaluated the potential toxicity of UDSCs according to the administration dosage and frequency using clinical pathological and histopathological analyses. We quantitatively assessed the in vivo distribution and retention period of UDSCs in major organs after single and repeated administration using human Alu-based qPCR analysis. We also conducted long-term monitoring for 26 weeks to assess the potential tumorigenicity.

**Results:**

Klotho-enhanced UDSCs exhibited excellent homing potential, and recovered Klotho expression in injured renal tissue. Toxicologically harmful effects were not observed in all mice after a single administration of UDSCs. It was also verified that repeated administration of UDSCs did not induce significant toxicological or immunological adverse effects in all mice. Single and repeated administrated UDSCs persisted in the blood and major organs for approximately 3 days and cleared in most organs, except the lungs, within 2 weeks. UDSCs that remained in the lungs were cleared out in approximately 4–5 weeks. There were no significant differences according to the variation of sex and administration frequency. The tumors were found in the intravenous administration group but they were confirmed to be non-human origin. Based on these results, it was clarified that UDSCs have no tumorigenic potential.

**Conclusions:**

Our results demonstrate that Klotho-enhanced UDSCs can be manufactured as cell therapeutics through an optimized GMP procedure, and they can be safely administered without causing toxicity and tumorigenicity.

**Supplementary Information:**

The online version contains supplementary material available at 10.1186/s13287-023-03595-y.

## Background

Urine-derived stem cells (UDSCs) were newly discovered and identified through research on a specific subpopulation of cells isolated from human urine [1–3]. These cells exhibit excellent stem cell properties such as clonogenicity, self-renewal capacity, multipotency, homing effect, and immune-modulatory capabilities [1, 3–7]. Previous studies have indicated that glomerular parietal epithelial cells within renal glomeruli can undergo an epithelial–mesenchymal transition (EMT) process and acquire stem cell phenotypes [8–10]. These cells are proposed as the origin of UDSCs, as they can be excreted from the body through urine under normal physiological conditions. Despite UDSCs being present in a small proportion of the overall urine-derived cells, they have been reported to play a crucial role in promoting tissue regeneration within their origin organ [9, 11–16].

Several studies have shown that UDSCs can express a variety of cell surface markers depending on the isolation methods and culture conditions [17–19]. However, it has been consistently characterized that UDSCs should predominantly express mesenchymal stem cells (MSCs)-related surface markers such as CD44, CD73, CD90, and CD105 [1, 3, 20–22]. Additionally, UDSCs distinctively express pluripotent stem marker, SSEA-4, and pericyte marker, CD146 [3, 5, 9, 19, 23]. UDSCs also possess the capability to differentiate into various cell types, such as adipocytes, osteocytes, chondrocytes, and smooth muscle cells [1, 3, 19, 23–25]. Moreover, due to their origin-specificity, UDSCs exhibit a higher efficiency in differentiating into cells that constitute renal tissues compared to typical MSCs [3, 5, 26].

Our latest study has revealed that UDSCs express Klotho, a kidney-specific therapeutic factor that is rarely found in other MSCs [27]. Klotho proteins play a protective role for kidneys, and it has been revealed that the blood level of Klotho gradually decreases according to the progression of chronic kidney disease (CKD) in patients [28–33]. Based on these findings, we proposed that UDSCs can be a novel therapeutic alternative to overcome the limitations of conventional therapies for various nephropathies including CKD. To ensure the clinical applicability of UDSCs, we have initially optimized the manufacturing procedure of a Good Manufacturing Practice (GMP)-grade UDSCs for the stable and massive production of safe cell therapeutics after collecting urine from the patient. Furthermore, we developed a cell culture method for UDSCs to enhance the expression of endogenous Klotho, which is a key therapeutic factor for CKD treatment.

Prior to clinical application, it is necessary to validate the potential risks and unintended effects of cells through non-clinical trials, since stem cells have distinctive properties such as self-renewal, differentiation, responses to the physiological environment, and migration to other sites after administration. In this study, we aim to evaluate the safety and biodistribution of clinical-grade UDSCs through non-clinical tests (Table 1) following the guidelines of Good Laboratory Practice (GLP). Our examinations include various aspects related to long-term survival and proliferation, non-target tissue distribution, undesired differentiation, heterologous tissue formation, tumor formation, and potential toxic reactions after UDSCs administration.

**Table 1:** Overview of non-clinical safety and biodistribution study design

Mouse strain	Dosages (male/female)	Route	Number of administrations	Observation periods (male/female)
*(1) Single- and repeated-dose toxicity*				
BALB/c nude mice (*n* = 40)	G1: Vehicle (5/5) G2: 5.0 × 10^5^ UDSCs/head (5/5) G3: 1.5 × 10^6^ UDSCs/head (5/5) G4: 4.5 × 10^6^ UDSCs/head (5/5)	IV	Single injection	2 weeks
BALB/c nude mice (*n* = 100)	G1: Vehicle (15/15) G2: 5.0 × 10^5^ UDSCs/head (10/10) G3: 1.5 × 10^6^ UDSCs/head (10/10) G4: 4.5 × 10^6^ UDSCs/head (15/15)	IV	Quadruple injection (1-week intervals)	3 weeks (Additional 2 weeks for recovery group)
*(2) Single- and repeated-dose biodistribution*				
BALB/c nude mice (*n* = 80)	4.5 × 10^6^ UDSCs/head	IV	Single injection	2 h (5/5) 1 day (5/5) 3 days (5/5) 1 week (5/5) 2 weeks (5/5) 4 weeks (5/5) 6 weeks (5/5) 8 weeks (5/5)
BALB/c nude mice (*n* = 50)	4.5 × 10^6^ UDSCs/head	IV	Triple injection (1-week intervals)	2 h (5/5) 1 day (5/5) 2 weeks (5/5) 4 weeks (5/5) 5 weeks (5/5)
*(3) Repeated-dose tumorigenicity*				
BALB/c nude mice (*n* = 80)	G1: Vehicle (10/10) G2: 2.0 × 10^6^ HT-1080/head (10/10) G3: 4.5 × 10^6^ UDSCs/head (10/10) G4: 4.5 × 10^6^ UDSCs/head (10/10)	SC, IV SC SC IV	Quadruple injection (1-week intervals)	26 weeks

*G* group, *IV* intravenous, *SC* subcutaneous, *HT-1080* human fibrosarcoma cell line

## Methods

### GMP manufacturing of UDSCs

In accordance with ethical standards and regulations, we obtained approval from the Korean Institutional Review Board (IRB) to collect human urine samples for this study. Fresh urine samples were collected from patients and transferred to the GMP facility. Urine-derived cells were then isolated following Standard Operating Procedure (SOP), which included a filtration, centrifugation, and washing process. In detail, the collected urine was filtered through a 100 µm cell strainer to remove impurities. The filtrate was then centrifuged 500 × g for 15 min. The supernatant was carefully aspirated, and the pellets were washed with DPBS containing 1% Antibiotic–Antimycotic (Gibco, Waltham, MA, USA) (repeated 3 times). Subsequently, the suspended cells were plated onto 24-well plates using a customized expansion medium (EX-medium). Seeding on a 24-well plate allows for the isolation of single UDSCs from non-stem cells present in urine, enabling high-purity UDSC colonies in GMP manufacture. The EX-medium was formulated by mixing DMEM and F12 media at a 3:1 ratio, and supplemented with 5% FBS, 1% Antibiotic–Antimycotic, 1% ITS-E (Biogems, Westlake Village, CA, USA), 0.25% Albumax™ II (Gibco, Waltham, MA, USA), 20 ng/mL EGF, 2 ng/mL bFGF, 2 nM Triiodothyronine (T3), 2 µM Retinyl acetate, 0.2 mM L-Ascorbic acid 2-phosphate, and 50 nM 25-Hydroxycholecalciferol. After 10–15 days, UDSC colonies were selectively collected and expanded, and UDSCs were cryopreserved at passage 3. For each experiment, UDSCs were further cultured using a customized Klotho activation medium (KL-medium), and harvested at passage 5. The KL-medium was formulated based on the EX-medium, with the addition of two supplements that can enhance the expression of Klotho in UDSCs. Such supplements can include a variety of growth factors, hormones, and small molecules associated with the biological activation of Klotho in vivo. Specific details regarding the supplements or their combinations used in this study have been omitted for commercial or patent-related reasons.

### Characterization of Klotho-enhanced UDSCs

The characterization of UDSCs was confirmed by trilineage differentiation (adipo-, osteo-, and chondrogenesis), flow cytometry (CD73, CD90, CD146, SSEA-4, CD31, and CD34), and western blotting (Klotho), using the methods previously described [27].

For adipogenic and osteogenic differentiation, UDSCs were seeded on a 6-well plate at 10,000 cells/cm^2^ and cultured in respective differentiation mediums for 21 days. Differentiation was verified using Oil Red O for adipogenesis and Alizarin Red S for osteogenesis. For chondrogenic differentiation, UDSCs were seeded at 10,000 cells/cm^2^ in an ultra-low-attachment 96-well plate (Corning, Corning, NY, USA) and the UDSCs spheroids were cultured for 21 days using the StemPro™ chondrogenesis differentiation kit (Gibco, Waltham, MA, USA). Differentiation was verified using Alcian Blue staining.

For surface marker analysis, UDSCs were stained with MSC (CD73, CD90), pericyte (CD146), pluripotent stem cell (SSEA4), endothelial (CD31), and hematopoietic (CD34) related markers (Additional file 1: Table S1). UDSCs are typically positive for CD73, CD90, CD146, and SSEA4; while, they are negative for CD31 and CD34. To evaluate the purity of clinical-grade UDSCs, we established criteria based on these known characteristics. High-purity UDSCs were defined by the expression of positive markers at 90% or higher and the absence of negative markers at less than 10%.

### Animal management

This study was conducted at a GLP-certified institute (Chemon, Gyeonggi-do, South Korea). 4-week-old male and female BALB/c nude (BALB/cSlc-nu/nu) mice were purchased from Japan SLC Inc. (Shizuoka, Japan). All purchased mice underwent thorough assessments, such as physical condition, body weight, general symptoms, and pathogen status, and no abnormalities were detected. All mice were randomly grouped for the experiments. The mice were identified using ear-punch and were housed under conditions of 22 ± 3 °C temperature and 55 ± 15% relative humidity. The examinations were initiated when the mice reached 6 weeks of age (male: 19.87–23.20 g, female: 17.36–20.66 g).

Mice were anesthetized using isoflurane delivered via precision vaporizer at each necropsy time point. For the collection of blood for clinical pathological examination, the abdominal aorta and portocaval vein of the mice were severed to ensure complete exsanguination while simultaneously euthanizing them. Subsequently, a comprehensive necropsy and histological analysis were performed, encompassing the entire body, subcutaneous tissue, head, thoracic, and abdominal cavities, as well as all organs. Detailed necropsy findings were systematically documented.

### Therapeutic potential of Klotho-enhanced UDSCs

To induce ischemia–reperfusion injury (IRI) in 8-week-old BALB/c nude mice, bilateral renal artery clamping was conducted for 30 min. 24 h post-IR injury, UDSCs were labeled with PKH26 Red Fluorescent Cell Linker Kit (Sigma-Aldrich, Burlington, MA, USA) and then intravenously administered via the tail vein at a dose of 2.0 × 10^6^ cells/head. Another 24 h later, the mice were euthanized, and the localization of UDSCs was detected by fluorescence microscopy.

One week post-IR injury, the mice were intravenously administered with UDSCs through the tail vein at a dose of 2.0 × 10^6^ cells/head. After an additional week, the mice were euthanized, and renal tissues were analyzed for Klotho protein expression. Immunofluorescence staining was performed using an anti-human Klotho monoclonal antibody (Cosmo Bio USA, Carlsbad, CA, USA). The obtained Klotho expression data were compared to control group.

### Experimental groups and UDSCs administration

In the toxicity tests, UDSCs were administered as single- and repeated-dose at three different concentrations: low (5.0 × 10^5^ cells/head), medium (1.5 × 10^6^ cells/head), and high (4.5 × 10^6^ cells/head). The cells were reconstituted with 200 μL of vehicle (normal saline) and administered via the tail vein at a 1 mL/min rate.

The cell concentrations were preliminarily determined based on dose range finding (DRF) tests. UDSCs were administered at various concentrations with repeated doses (double injection, 1-week intervals), and then all organs were analyzed for absolute and relative weights, histological changes, and necropsy. In our DRF results, the lowest concentration that showed significant histopathological changes was determined as the high dose for this toxicity test. Furthermore, considering our intended clinical administration dose, the experimental design involved establishing a roughly tenfold difference between the highest and lowest doses, strategically aimed at exploring the safe dosage range. Therefore, the differences between each concentration group were set at approximately 3-folded.

In the tumorigenicity test, high-dose of UDSCs were administered via subcutaneous and intravenous routes, and a human fibrosarcoma cell line was injected as a positive control. The in vivo distribution was assessed at different necropsy points after single and repeated administration. Additional information for each examination is summarized in Table 1. The results from all mice used in the experiments were included in the dataset.

### Observation

All mice were monitored at least once daily for mortality and general symptoms, including onset, types, and severity. Body weight is a general parameter for ensuring dosage appropriateness and accurate representation of intended exposure levels. Also, changes in weight can be indicative of the overall health status of the mice during the experiment period. Therefore, body weight was measured at least once a week after UDSCs administration.

### Clinical-pathological analysis

0.3 mL of fresh urine was collected from all mice at the final necropsy time point. Urinary analysis was performed using a Multistix 10SG reagent strip (Siemens Healthineers, Erlangen, Germany). The mice were then anesthetized with isoflurane inhalation, and the blood sample was collected from the retro-orbital sinus. For hematological analysis, a 0.3 mL blood sample was collected into a 3 mL CBC bottle (BD Biosciences, California, USA) containing EDTA-3K, and measured using ADVIA 2120 automated hematology analyzer (Siemens Healthineers, Germany). For biochemical analysis, a 0.5 mL of blood sample was collected into a 5 mL SST™ II Advance vacutainer (BD Biosciences, California, USA) containing a clot activator, and incubated at room temperature for 15–20 min to allow clotting. Subsequently, the serum was obtained through centrifugation at 3000 rpm for 10 min, and measured using AU680 biochemical analyzer (Beckman Coulter, California, USA).

### Histopathological analysis

All organs, including the injection sites, were visually examined to evaluate structural and functional abnormalities and pathological changes. Absolute weights of major organs, including the brain, lungs, heart, liver, and kidneys, were measured using an electronic balance, and relative weights of the organs to body weight were calculated. For histopathological analysis, all organs were fixed in a 10% Neutral Buffered Formalin (NBF) solution and the tissue sections were prepared. Histopathological findings followed the International Harmonization of Nomenclature and Diagnostic Criteria (INHAND), and referred to the Standardized System of Nomenclature and Diagnostic Criteria (SSNDC) by the American Society of Toxicologic Pathology (STP), as necessary.

### Lymphocyte phenotype analysis

To isolate splenocytes, the spleen was preserved in a tube containing 5 mL of RPMI1640 medium and homogenized using a syringe plunger. The cells were then centrifuged, washed, and resuspended with DPBS. The samples were labeled with specific antibodies for lymphocyte subsets (CD3, CD4, CD8, CD45R, and CD49b) and analyzed using a BD Accuri™ C6 flow cytometer (BD Biosciences, California, USA).

### Human Alu-based qPCR analysis

Genomic DNA (gDNA) was extracted from major organs using the G-DEX IIC genomic DNA extraction kit (Intron Biotechnology, Seoul, South Korea). The extracted gDNA was quantitatively analyzed using a specific primer/probe set designed for the human genome: Human Alu Forward primer (5′-TTA GCC GGA CGT AGT GGC-3′), Human Alu Reverse primer (5′-GCA ATC TCG GCT CAC TGC AA-3′), Human Alu Probe (5′-FAM-AGC TAC TCG GGA GGC TGA GGC AGG A-BHQ1-3′). Linearity, limit of detection and quantification, accuracy, precision, and specificity of the qPCR analysis were validated at least 3 times prior to the examination.

### Statistical analysis

The statistical analysis was performed using Provantis™ (Instem, Stafford, UK), and the significance level was set at *p* < 0.05. ANOVA followed by Dunnett’s post hoc test was used for comparing three or more experimental groups; while, post hoc pairwise comparisons using t test were used for comparing independent pairs of groups.

## Results

### Characterization of Klotho-enhanced UDSCs

All the procedures for UDSCs isolation and cultivation were performed in a GMP facility. The UDSCs isolated from fresh urine exhibited a rice grain-like morphology during the initial stages of cell attachment. After approximately 10–15 days, the UDSCs formed colonies with clear and round borders, which are distinct from other cells. Then, the UDSC colonies were selectively collected and enzymatically passaged, and they proliferated in a spindle-shaped morphology similar to fibroblasts. Several analyses were performed for the characterization of UDSCs.

**Fig. 1:**
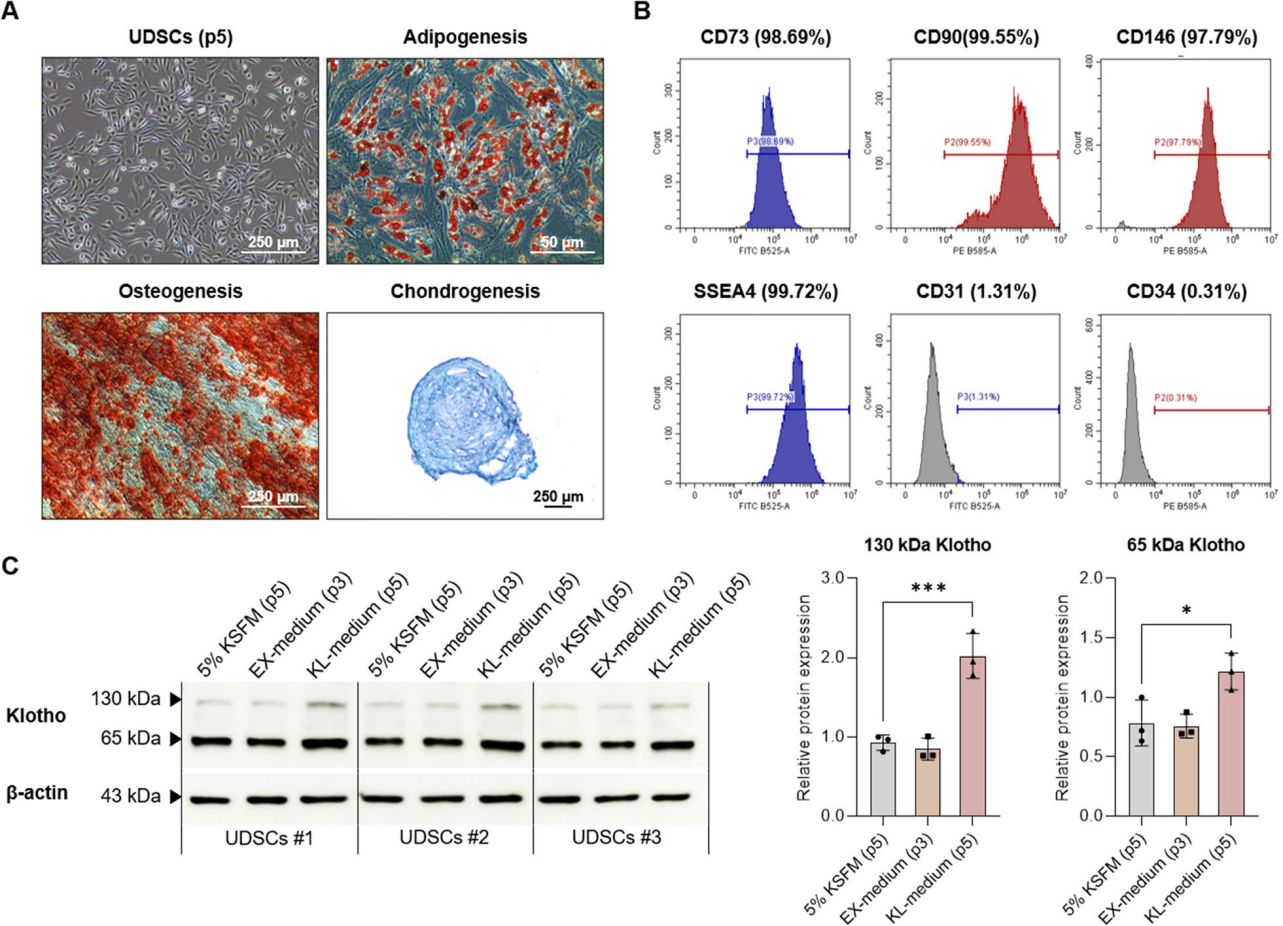
Characterization of Klotho-enhanced UDSCs. In these experiments, all UDSCs from different donors were manufactured in a GMP facility. **A** Multi-differentiation capability was confirmed by Oil Red O, Alizarin Red S, and Alcian Blue staining. **B** Cell surface marker expression was analyzed by flow cytometry at passage 5. **C** An increase in Klotho protein expression after culturing with KL-medium was confirmed by western blotting. Full-length original blot images are presented in Additional file 2: Figure S1. Results were shown as the mean ± SEM. *p < 0.05, ***p < 0.001 vs. 5% KSFM (p5). KSFM, keratinocyte serum-free medium; IRI, Ischemia–reperfusion injury

First, the UDSCs at passage 5 were induced for trilineage differentiation to assess their potential to differentiate into adipocytes, osteocytes, and chondrocytes. The differentiation capability into these three lineages was confirmed by staining (Fig. 1A).

Second, flow cytometry analysis was performed and identified that the UDSCs were positively expressed MSC-related surface markers, such as CD44 and CD73, with over 90% expression. Additionally, the UDSCs were also strongly expressed SSEA-4, a pluripotent stem cells (PSCs) marker, and CD146, a pericyte marker. However, the UDSCs negatively expressed CD31, an endothelial cell marker, and CD34, a hematopoietic stem cell marker (Fig. 1B).

Last, western blotting was performed to analyze the klotho expression of UDSCs. Here we compared Klotho expression between UDSCs expanded with a customized expansion medium (EX-medium) until passage 3 and UDSCs expanded an additional two more passages with a customized Klotho activation medium (KL-medium) after initial cultivation with EX-medium. Furthermore, UDSCs cultured with commonly known UDSCs culture media (KSFM containing 5% FBS) were used as a control. As a result, it was revealed that KL-medium significantly enhanced the expression level of both 130 kDa and 65 kDa forms of Klotho in UDSCs (Fig. 1C).

### Therapeutic potentials of Klotho-enhanced UDSCs

**Fig. 2:**
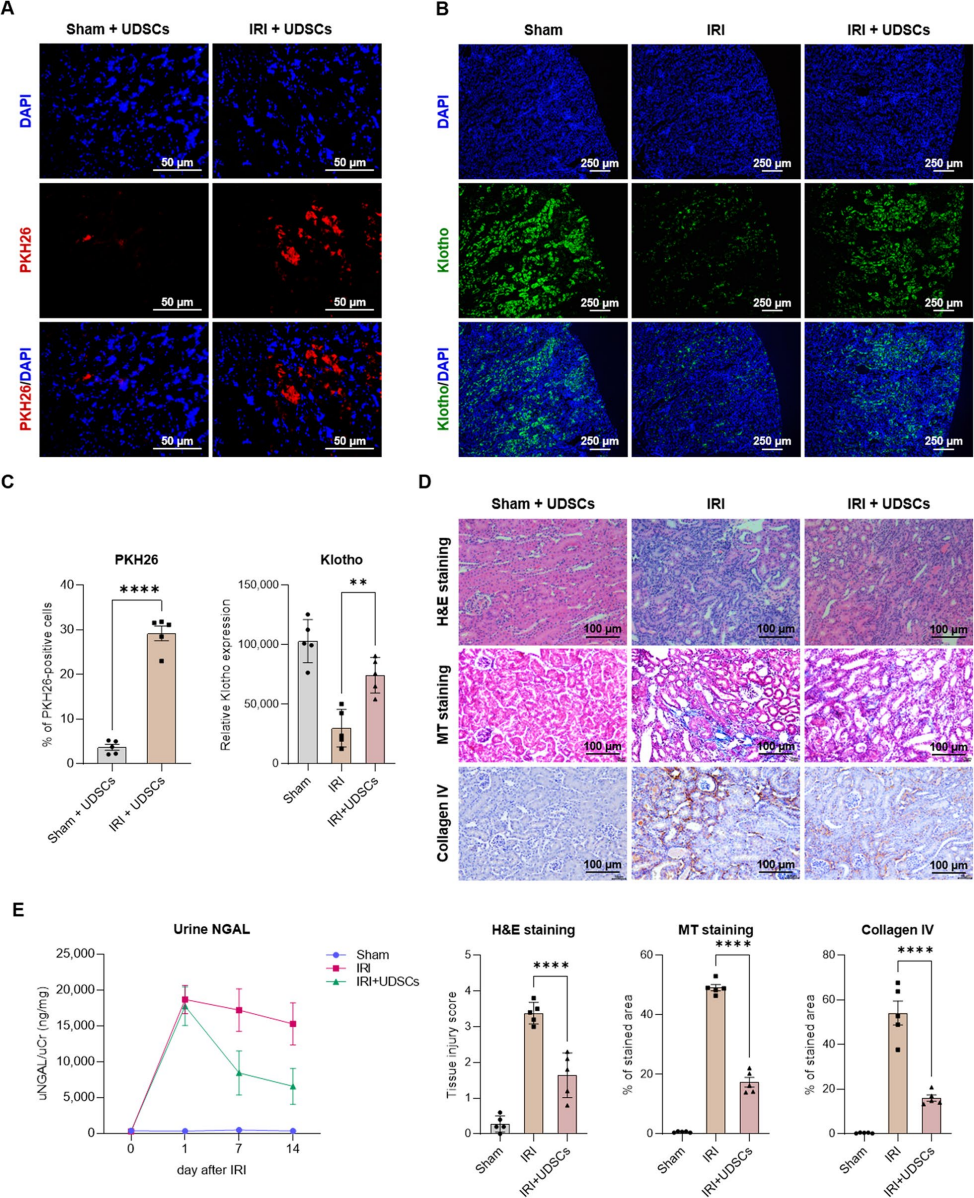
Therapeutic potentials of Klotho-enhanced UDSCs. **A** Klotho-enhanced UDSCs (PKH26: Red) were intravenously administered to both wild-type (Sham) and ischemia–reperfusion injury (IRI) groups. **B** Klotho expression (Klotho: Green) was detected in the renal cortex region after Klotho-enhanced UDSCs administration. **C** The images of A and B have been quantitatively analyzed. **D** Tissue injury was assessed using H&E staining. Renal fibrosis was measured using MT staining and type IV Collagen expression. **E** Kidney injury was represented by urine NGAL levels and was normalized using urine creatinine levels. Results were shown as the mean ± SEM. **p < 0.01, ****p < 0.0001. H&E, hematoxylin and eosin; MT, Masson’s trichrome; NGAL, neutrophil gelatinase associated lipocalin

We investigated the migration and localization capabilities of Klotho-enhanced UDSCs. For the homing analysis, ischemia–reperfusion injury (IRI) was induced in the kidneys of BALB/c nude mice. 24 h post-IRI, we intravenously administered a single dose of PKH26-labeled UDSCs (2.0 × 10^6^ cells/mouse) via the tail vein. Another 24 h later, the localization of UDSCs was observed using fluorescence microscopy. Our results showed that the UDSCs significantly localized to the renal tissues of the IRI group, as compared to the sham group (Fig. 2A, C).

Furthermore, we conducted experiments to assess the potential of Klotho-enhanced UDSCs with the ability to recover Klotho protein. One week post-IR injury, we administered a single dose of UDSCs (2.0 × 10^6^ cells/mouse) intravenously. After an additional week, the Klotho expression levels in renal tissues were assessed using immunofluorescence staining. Our findings indicated a significant decrease in Klotho expression from the renal cortex due to the IR injury. Nevertheless, the administration of Klotho-enhanced UDSCs effectively recovers the endogenous Klotho protein expression in the renal tissues (Fig. 2B, C).

Our previous research demonstrated that Klotho secreted from UDSCs inhibited fibrosis of tubular epithelial cells. We evaluated the severity of kidney injury and fibrosis using immunohistochemistry staining. The administration of Klotho-enhanced UDSCs ameliorated tissue injury, and led to a significant reduction in fibrosis progression, especially the secretion of collagen IV within the renal tissue (Fig. 2D).

Urine Neutrophil Gelatinase Associated Lipocalin (uNGAL) is widely recognized as a highly sensitive specific marker to the diagnosis of acute kidney injury (AKI). IR injury initiated a pronounced increase in uNGAL levels. The administration of Klotho-enhanced UDSCs significantly downregulated the elevation of uNGAL level (Fig. 2E).

### Toxicity of UDSCs following single and repeated administration

**Fig. 3:**
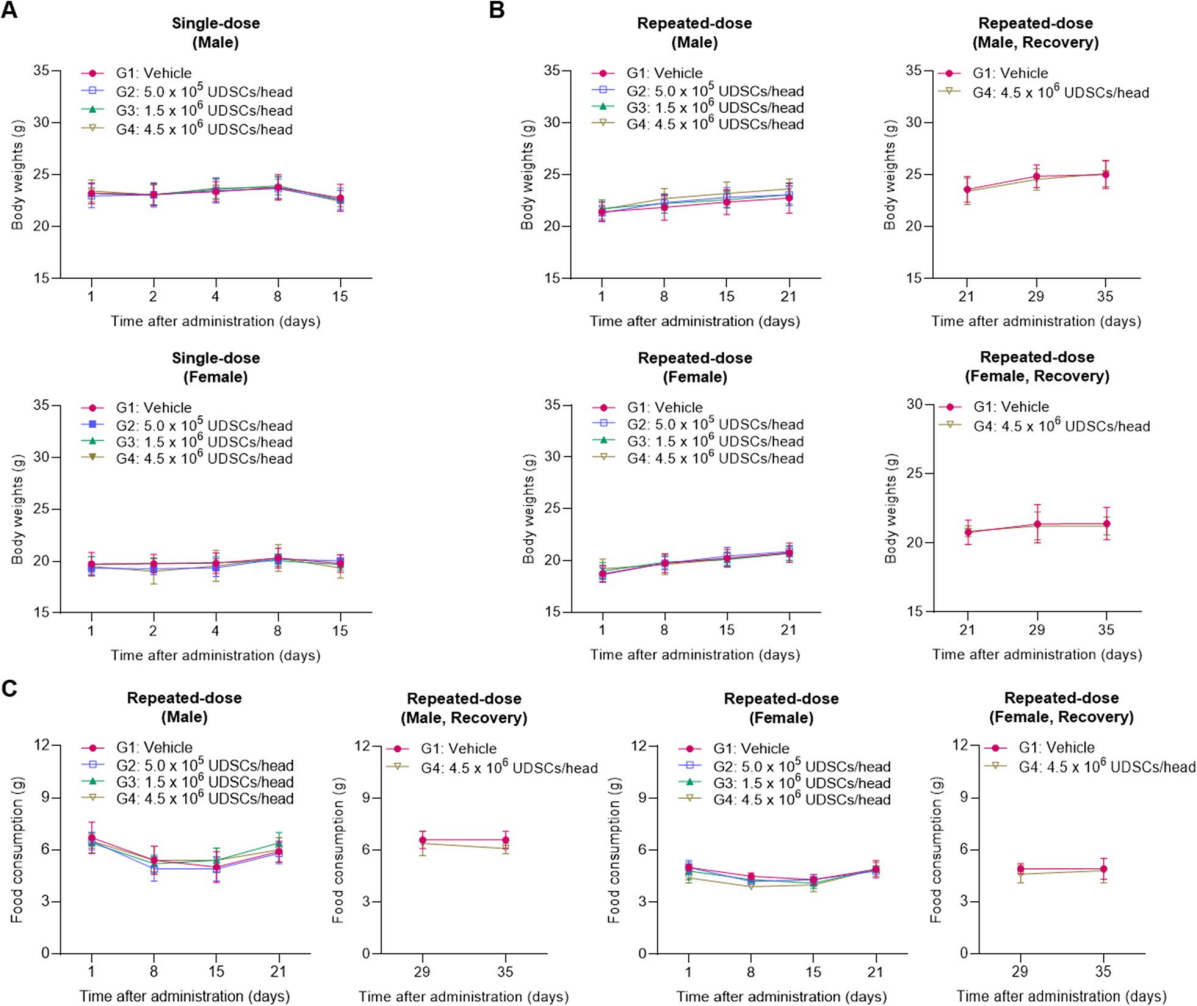
The body weight and food consumption changes following a single and repeated administration. **A**, **B** Body weight changes after a single and repeated intravenous administration were assessed. **C** Food consumption after repeated intravenous administration was assessed. An additional 2-week recovery period in the vehicle control (G1) and high-dose group (G4) was investigated. Results were shown as the mean ± SEM

We investigated the potential toxicity of GMP-manufactured UDSCs after in vivo administration in BALB/c nude mice. First, a single intravenous administration of UDSCs was performed with three different concentrations (G2: 5 × 10^5^, G3: 1.5 × 10^6^, G4: 4.5 × 10^6^) in both male and female mice. Normal saline was administered as a vehicle control group (G1). During the 2-week observation period, no adverse effects related to UDSCs on mortality, general symptoms, body weight changes, or necropsy findings were observed in any of the experimental groups (Fig. 3A). Based on the result, the Approximate Lethal Dose (ALD) was determined to be greater than the high-dose of 4.5 × 10^6^ cells/head for both male and female mice.

Next, a repeated intravenous administration (quadruple injection, 1-week intervals) of UDSCs was performed with three different concentrations (G2: 5 × 10^5^, G3: 1.5 × 10^6^, G4: 4.5 × 10^6^), and an additional 2-week recovery period in the vehicle control (G1) and the high-dose group (G4) was investigated. During the 5-week examination period, no adverse effects related to UDSCs were observed in terms of mortality, general symptoms, body weight changes, food consumption, urinalysis, or ophthalmological examinations (Fig. 3B, C).

Meanwhile, necropsy and histopathological findings revealed a few instances of organ hypertrophy, atrophy, and malformation in both male and female mice. However, these results showed no dose–response relationship, were infrequent, or were associated with changes in reproductive cycles. Compared to the vehicle control group (G1), these abnormalities were clarified to be naturally occurring variations in BALB/c nude mice (Table 2).

**Table 2:** Necropsy findings and histopathological diagnosis following repeated administration of UDSCs in toxicity test

Organ	Necropsy findings	Group (#Number)	Histopathological diagnosis
Spleen	Enlarged	G1 (#12)	Increased extramedullary hematopoiesis
Epididymis	Small	G2 (#24) G4 (#43,44)	Atrophy (#24, 43, 44), Luminal reduced sperm (#24)
Kidney	Abnormal Shape	G4 (#50)	Unilateral hypoplasia
	Discoloration		
Submandibular lymph node	Enlarged	G2 (#18)	Increased cellularity of plasma cell
Testis	Small	G2 (#24), G4 (#43, 44)	Tubular degeneration/ atrophy
Uterus	Clear retention	G1 (#55, 56, 65) G2 (#73) G3 (#76, 79, 81) G4 (#86, 87, 89, 90, 95, 100)	Luminal dilatation
Thoracic spine	Abnormal Shape	G1 (#3)	No correlated lesion

Male: #1–#50, Female: #51–#100

In the results of organ weights, both absolute and relative lung weights significantly increased in all UDSCs-administered groups (G2–G4). This increase in lung weight demonstrated a dose–response relationship and was consequently determined to be an effect of UDSCs administration. However, no histopathological changes in the lungs were observed. Significant decreases in absolute brain weight and relative weight of the left ovary were observed in the high-dose female group (G4). However, these were not accompanied by abnormal behavior or any histopathological abnormalities. Therefore, the weight changes in organs, excluding the lungs, were determined to be unrelated to the UDSCs (Table 3).

**Table 3:** Absolute and relative organ weight changes following repeated administration of UDSCs

Sex	Major organs	Observation period (week 3)				Recovery period (week 5)	
		G1 (*n* = 10)	G2 (*n* = 10)	G3 (*n* = 10)	G4 (n = 10)	G1 (*n* = 10)	G4 (*n* = 10)
Male	Terminal BW	20.93 g	21.44 g	21.49 g	22.24 g **	23.21 g	23.01 g
	Brain	0.4042 g (1.9327%)	0.4093 g (1.9115%)	0.4026 g (1.8759%)	0.4160 g (1.8721%)	0.4125 g (1.7785%)	0.4058 g (1.7665%)
	Heart	0.1522 g (0.7284%)	0.1512 g (0.7052%)	0.1557 g (0.7248%)	0.1547 g (0.6961%)	0.1556 g (0.6712%)	0.1568 g (0.6813%)
	Kidney (Left)	0.2044 g (0.9771%)	0.2099 g (0.9787%)	0.2044 g (0.9509%)	0.2143 g (0.9636%)	0.2232 g (0.9603%)	0.2280 g (0.9912%)
	Kidney (Right)	0.2051 g (0.9801%)	0.2071 g (0.9665%)	0.2072 g (0.9649%)	0.2195 g (0.9867%)	0.2207 g (0.9486%)	0.2307 g (1.0028%)
	Liver	1.3912 g (6.6452%)	1.3741 g (6.4012%)	1.3413 g (6.2375%) **	1.4862 g (6.6827%)	1.4916 g (6.4139%)	1.4788 g (6.4310%)
	Lung	0.1405 g (0.6711%)	0.1488 g (0.6940%)	0.1567 g * (0.7295%) *	0.1816 g *** (0.8162%) ***	0.1409 g (0.6068%)	0.1525 g (0.6634%)
	Spleen	0.0939 g (0.4488%)	0.0966 g (0.4512%)	0.0957 g (0.4442%)	0.1083 g (0.4865%)	0.1363 g (0.5851%)	0.1106 g (0.4819%)
	Testis (Left)	0.0792 g (0.3782%)	0.0767 g (0.3579%)	0.0799 g (0.3723%)	0.0742 g (0.3328%)	0.0871 g (0.3754%)	0.0880 g (0.3834%)
	Testis (Right)	0.0803 g (0.3836%)	0.0759 g (0.3544%)	0.0830 g (0.3861%)	0.0827 g (0.3714%)	0.0909 g (0.3922%)	0.0886 g (0.3855%)
Female	Terminal BW	19.27 g	19.50 g	19.27 g	19.09 g	19.80 g	19.77 g
	Brain	0.4317 g (2.2446%)	0.4254 g (2.1829%)	0.4225 g (2.1952%)	0.4144 g * (2.1718%)	0.4252 g (2.1495%)	0.4192 g (2.1213%)
	Heart	0.1373 g (0.7127%)	0.1388 g (0.7120%)	0.1329 g (0.6901%)	0.1437 g (0.7533%)	0.1300 g (0.6566%)	0.1290 g (0.6527%)
	Kidney (Left)	0.1535 g (0.7977%)	0.1571 g (0.8054%)	0.1518 g (0.7877%)	0.1563 g (0.8195%)	0.1500 g (0.7582%)	0.1531 g (0.7739%)
	Kidney (Right)	0.1576 g (0.8186%)	0.1564 g (0.8026%)	0.1520 g (0.7889%)	0.1568 g (0.8213%)	0.1556 g (0.7864%)	0.1512 g (0.7646%)
	Liver	1.1426 g (5.9226%)	1.1994 g (6.1551%)	1.1544 g (5.9930%)	1.1506 g (6.0201%)	1.1882 g (5.9961%)	1.1913 g (6.0289%)
	Lung	0.1376 g (0.7142%)	0.1511 g * (0.7747%)	0.1535 g * (0.7980%) **	0.1753 g *** (0.9185%) ***	0.1332 g (0.6717%)	0.1418 g (0.7175%)
	Ovary (Left)	0.0028 g (0.0144%)	0.0026 g (0.0135%)	0.0026 g (0.0133%)	0.0028 g 0.0145%)	0.0028 g (0.0141%)	0.0021 g (0.0105%) *
	Ovary (Right)	0.0025 g (0.0128%)	0.0030 g (0.0154%)	0.0026 g (0.0134%)	0.0027 g (0.0143%)	0.0028 g (0.0139%)	0.0024 g (0.0122%)
	Spleen	0.1121 g (0.5812%)	0.1192 g (0.6112%)	0.1170 g (0.6064%)	0.1258 g (0.6578%)	0.1339 g (0.6729%)	0.1134 g (0.5734%)

*BW* body weight

Results were shown as the mean absolute BW (mean relative BW). *p < 0.05, **p < 0.01, ***p < 0.001 vs. vehicle control group (G1)

In the hematological analysis, significant decreases in PLT, NEU, EOS, and an increase in MPV were observed in a dose–response manner in the medium- and high-dose groups (G3, G4). These changes were subsequently determined to be effects of the UDSCs administration. However, no histopathological changes were observed in related organs, such as the spleen, liver, and bone marrow, during the histopathological examination. This indicates that there were no toxicologically harmful effects (Table 4).

**Table 4:** Hematological analysis following repeated administration of UDSCs

Sex	Parameters	Observation period (week 3)				Recovery period (week 5)	
		G1 (*n* = 10)	G2 (*n* = 10)	G3 (*n* = 10)	G4 (*n* = 10)	G1 (*n* = 5)	G4 (*n* = 5)
Male	RBC (10^6^/μL)	9.88 ± 0.35	9.71 ± 0.31	9.61 ± 0.33	9.60 ± 0.27	9.35 ± 0.28	9.21 ± 0.27
	HGB (g/dL)	15.9 ± 0.5	15.8 ± 0.6	15.5 ± 0.6	15.6 ± 0.5	14.8 ± 0.5	14.4 ± 0.4
	PLT (10^3^/μL)	1027.3 ± 35	914.9 ± 137.1	665.4 ± 180.7***	376.6 ± 60.5***	1032.8 ± 102.7	1141.8 ± 94.3
	MPV (fL)	6.30 ± 0.24	6.55 ± 0.59	6.62 ± 0.47	6.89 ± 0.37 *	6.00 ± 0.00	6.04 ± 0.19
	WBC (10^3^/μL)	2.21 ± 0.81	1.69 ± 0.71	1.64 ± 1.07	1.38 ± 0.42	1.44 ± 0.74	0.88 ± 0.21
	NEU (10^3^/μL)	1.00 ± 0.37	0.74 ± 0.23	0.71 ± 0.41	0.53 ± 0.15**	0.79 ± 0.51	0.47 ± 0.13
	LYM (10^3^/μL)	1.06 ± 0.45	0.84 ± 0.47	0.81 ± 0.59	0.76 ± 0.30	0.56 ± 0.25	0.36 ± 0.09
	MONO (10^3^/μL)	0.07 ± 0.04	0.06 ± 0.03	0.06 ± 0.06	0.05 ± 0.02	0.05 ± 0.03	0.02 ± 0.01
	EOS (10^3^/μL)	0.06 ± 0.02	0.04 ± 0.01	0.04 ± 0.02	0.03 ± 0.01*	0.03 ± 0.02	0.02 ± 0.01
Female	RBC (10^6^/μL)	9.40 ± 0.31	9.37 ± 0.27	9.36 ± 0.35	9.37 ± 0.21	9.36 ± 0.37	9.34 ± 0.23
	HGB (g/dL)	15.4 ± 0.5	15.2 ± 0.5	15.4 ± 0.5	15.3 ± 0.2	14.9 ± 0.5	14.8 ± 0.6
	PLT (10^3^/μL)	1041.4 ± 87.4	918.4 ± 201.0	767.7 ± 239.4***	486.5 ± 167.1***	901.4 ± 167.2	1044.2 ± 39.1
	MPV (fL)	6.34 ± 0.39	6.44 ± 0.36	6.68 ± 0.98	6.55 ± 0.54	5.84 ± 0.18	5.80 ± 0.19
	WBC (10^3^/μL)	2.10 ± 0.99	2.97 ± 1.07	2.17 ± 0.90	1.99 ± 0.86	0.98 ± 0.33	1.50 ± 0.40
	NEU (10^3^/μL)	0.88 ± 0.33	1.02 ± 0.36	0.83 ± 0.24	0.84 ± 0.35	0.44 ± 0.15	0.75 ± 0.19
	LYM (10^3^/μL)	1.08 ± 0.60	1.74 ± 0.65	1.20 ± 0.64	1.02 ± 0.55	0.47 ± 0.16	0.67 ± 0.29
	MONO (10^3^/μL)	0.07 ± 0.07	0.11 ± 0.06	0.07 ± 0.03	0.06 ± 0.03	0.02 ± 0.01	0.03 ± 0.01
	EOS (10^3^/μL)	0.05 ± 0.02	0.07 ± 0.02	0.05 ± 0.01	0.05 ± 0.02	0.04 ± 0.01	0.04 ± 0.01

*EOS* eosinophils, *HGB* hemoglobin, *LYM* lymphocytes, *MONO* monocytes, *MPV* mean platelet volume, *NEU* neutrophils, *PLT* platelet count, *RBC* red blood cell, *WBC* white blood cell

In the blood biochemistry analysis, a significant increase in TCHO was observed in the medium- and high-dose male groups (G3, G4). This increase demonstrated a dose–response relationship and was considered to be an effect of UDSCs administration. However, no histopathological changes were observed in related organs, such as the liver and kidneys, during the histopathological examination. And a significant decrease in CPK was observed in the high-dose group (G4) which also showed a dose–response relationship. However, general symptoms, including body weight loss, and histopathological findings were not observed in related organs, such as the thyroid gland. Although the AST and ALT were significantly decreased in all UDSCs-administered female groups (G2–G4), this was a result of slightly higher levels in the vehicle control group (G1) and was considered unrelated to UDSCs administration (Table 5).

**Table 5:** Clinical biochemistry analysis following repeated administration of UDSCs

Sex	Parameters	Observation period (week 3)				Recovery period (week 5)	
		G1 (*n* = 10)	G2 (*n* = 10)	G3 (*n* = 10)	G4 (*n* = 10)	G1 (*n* = 5)	G4 (*n* = 5)
Male	AST (IU/L)	68.7 ± 6.8	82.3 ± 33.7	61.5 ± 3.7	59.7 ± 4.8	69.9 ± 6.5	70.9 ± 2.9
	ALT (IU/L)	32.7 ± 4.6	37.3 ± 17.9	28.1 ± 2.8	30.4 ± 3.7	35.2 ± 5.3	31.1 ± 2.5
	ALP (IU/L)	122.6 ± 15.1	130.7 ± 7.1	128.8 ± 8.2	125.1 ± 13.0	116.4 ± 14.1	122.9 ± 8.9
	CPK (IU/L)	138.9 ± 54.6	131.5 ± 71.9	94.2 ± 45.4	70.5 ± 17.9 *	82.8 ± 25.1	93.6 ± 12.7
	GLU (mg/dL)	237.6 ± 47.5	252.9 ± 45.9	247.5 ± 28.2	252.5 ± 40.9	250.6 ± 64.2	260.3 ± 41.8
	TCHO (mg/dL)	95.7 ± 5.3	100.9 ± 9.7	108.6 ± 4.5 **	108.1 ± 9.4 **	107.6 ± 10.2	103.2 ± 8.3
	TG (mg/dL)	85.6 ± 21.4	85.3 ± 22.0	79.4 ± 12.8	77.2 ± 22.3	75.6 ± 19.4	77.8 ± 15.7
	TP (g/dL)	4.36 ± 0.21	4.49 ± 0.21	4.55 ± 0.23	4.50 ± 0.18	4.46 ± 0.23	4.44 ± 0.18
	ALB (g/dL)	2.25 ± 0.09	2.32 ± 0.10	2.28 ± 0.08	2.27 ± 0.07	2.22 ± 0.11	2.24 ± 0.09
	BUN (mg/dL)	19.1 ± 3.2	21.0 ± 2.9	20.2 ± 1.8	18.5 ± 2.2	20.1 ± 4.1	23.5 ± 1.4
	CRE (mg/dL)	0.27 ± 0.02	0.29 ± 0.03	0.27 ± 0.02	0.25 ± 0.03	0.25 ± 0.03	0.26 ± 0.02
Female	AST (IU/L)	72.6 ± 6.8	62.7 ± 9.1 **	60.7 ± 5.6 **	59.9 ± 5.7 ***	96.4 ± 31.6	73.5 ± 6.8
	ALT (IU/L)	28.8 ± 4.0	24.0 ± 3.2 **	23.0 ± 3.4 **	24.0 ± 2.7 **	31.9 ± 7.7	27.7 ± 4.3
	ALP (IU/L)	118.3 ± 7.7	113.4 ± 10.0	119.0 ± 10.0	115.3 ± 13.5	116.2 ± 16.2	114.7 ± 8.1
	CPK (IU/L)	123.1 ± 54.4	95.9 ± 44.8	80.3 ± 34.2	68.2 ± 18.5*	69.4 ± 17.7	58.6 ± 7.3
	GLU (mg/dL)	221.1 ± 30.2	245.1 ± 41.5	231.7 ± 57.8	238.1 ± 41.4	296.5 ± 54.0	239.0 ± 35.7
	TCHO (mg/dL)	86.4 ± 9.4	88.5 ± 6.9	85.8 ± 4.9	90.4 ± 6.3	79.2 ± 7.8	86.2 ± 6.7
	TG (mg/dL)	72.0 ± 16.8	73.1 ± 15.4	70.8 ± 13.3	63.8 ± 10.1	78.2 ± 11.0	68.8 ± 9.0
	TP (g/dL)	4.53 ± 0.25	4.49 ± 0.33	4.41 ± 0.13	4.54 ± 0.21	4.48 ± 0.31	4.53 ± 0.22
	ALB (g/dL)	2.47 ± 0.09	2.44 ± 0.09	2.45 ± 0.06	2.47 ± 0.08	2.42 ± 0.09	2.43 ± 0.09
	BUN (mg/dL)	18.5 ± 1.8	17.8 ± 2.0	18.1 ± 2.3	18.9 ± 2.0	20.4 ± 4.8	19.2 ± 2.8
	CRE (mg/dL)	0.25 ± 0.02	0.25 ± 0.02	0.24 ± 0.03	0.24 ± 0.01	0.28 ± 0.04	0.24 ± 0.02

*ALB* albumin, *ALP* alanine aminotransferase, *AST* aspartate aminotransferase, *BUN* blood urea nitrogen, *CPK* creatinine phosphokinase, *CRE* creatinine, *GLU* glucose, *TCHO* total cholesterol, *TG* triglyceride, *TP* total protein

**Fig. 4:**
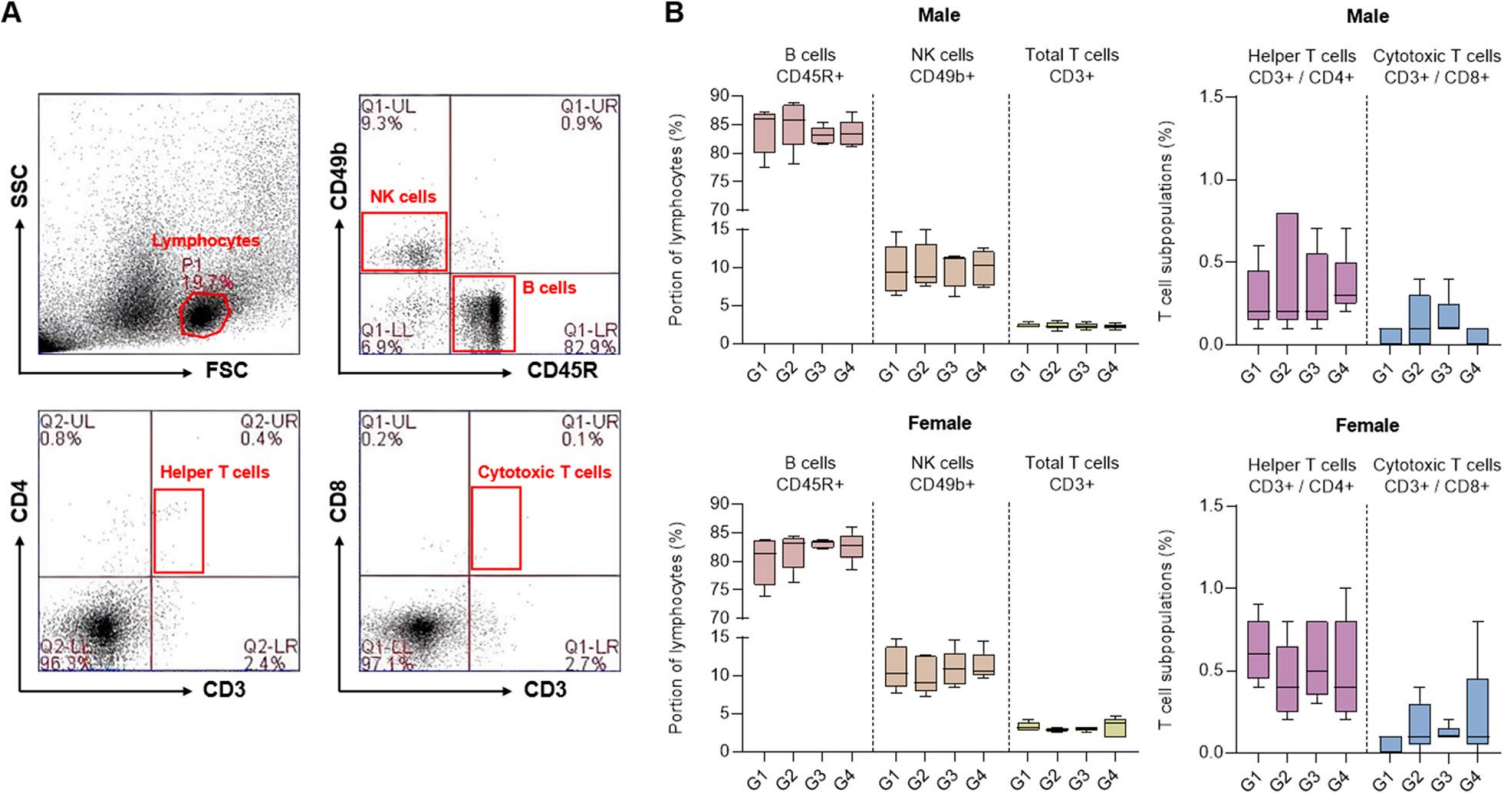
Lymphocyte phenotype and T cell subpopulation analysis following repeated administration of UDSCs. **A** Representative gating strategy: Lymphocytes were first gated based on forward scatter (FSC) versus side scatter (SSC), and CD3^+^, CD4^+^, CD8^+^, CD45R^+^, and CD49b^+^ cells were identified from the lymphocyte subset. **B** Both male and female mice showed no significant changes in lymphocyte and T cell subpopulation. Results were shown as the mean ± SEM

To assess the immunotoxicity after UDSCs administration, the proportions of CD45R^+^ B cells, CD49b^+^ NK cells, and CD3^+^ T cells within the spleen were measured by flow cytometry (Fig. 4A). The results showed that no significant changes were observed in the proportions of lymphocytes in any of the UDSCs-administered groups (G2–G4), for both male and female mice. Additionally, there were also no significant variations in the ratios of CD3^+^ total T cell subpopulations, CD3^+^/CD4^+^ helper T cells, and CD3^+^/CD8^+^ cytotoxic T cells. In conclusion, it can be inferred that the repeated administration of UDSCs did not exhibit immunotoxicity leading to adverse immune reactions, such as unexpected activations of lymphocyte subsets (Fig. 4B).

### Validation of human Alu-based qPCR quantification method

The in vivo distribution of UDSCs was quantitatively measured using qPCR analysis with primers and probes targeting the human-specific Alu (hAlu) genes. To evaluate the sensitivity and efficiency of the hAlu primers and probes, we validated the linearity, limit of detection, quantification, accuracy, precision, and specificity.

In the linearity test, the coefficient of determination (*R*^2^) values were confirmed to be ≥ 0.990 or higher in three repeated individual experiments. The minimum concentration that exhibited a significant difference in Ct values compared to the no template control (NTC) was confirmed to be 1 pg. Accordingly, the limit of detection (LOD) and lower limit of quantification (LLOQ) was determined to be 1 pg and 10 pg, respectively. In the accuracy test, the three repeated measurements of standard samples with known concentrations were close to the true values. The accuracy results showed a percent analytical recovery (%AR) within the range of 80–120% and a relative standard deviation (%RSD) within 20%. Also, the precision results showed %RSD values within 20% for repeated measurements by the same analyst across three or more concentrations. In the specificity test, the human genomic DNA of UDSCs was specifically detected under the conditions where it coexisted with the gDNA of target organs from mice. Based on these results, qPCR analysis using the hAlu genes was verified as a specific and accurate method for the quantification of UDSCs distribution (data were not shown).

### Biodistribution of UDSCs following a single administration

The distribution patterns in major organs were evaluated at 2 h, 1 day, 3 days, 1 week, 2 weeks, 4 weeks, 6 weeks, and 8 weeks after a single intravenous administration of UDSCs into BALB/c nude mice.

**Fig. 5:**
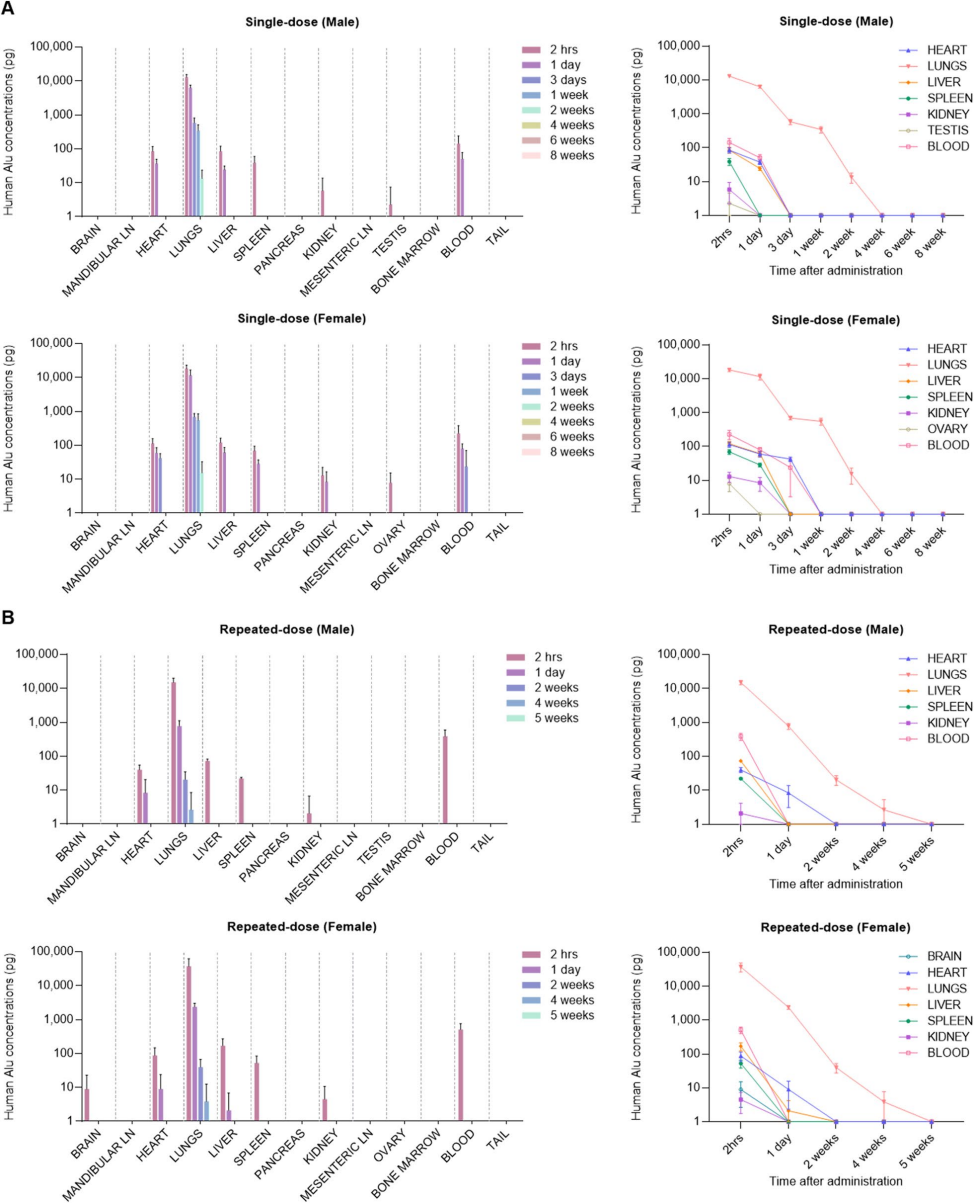
In vivo distribution following single and repeated administration of UDSCs. **A**, **B** After single and repeated administration of UDSCs, the hAlu-based qPCR analysis was conducted on major organs at various time points. The concentration of UDSCs was estimated using a validated quantification formula (data were not shown). Results were shown as the mean ± SEM. LOD = 1 pg. LLOQ = 10 pg. LN, lymph node

At the 2-h time point, the hAlu genes were detected in the heart, lungs, liver, spleen, and blood of all mice. Furthermore, in a subset of mice, the hAlu genes were detected in the kidneys (males: 2 out of 5, females: 4 out of 5), testes (males: 1 out of 5), and ovaries (females: 3 out of 5). In contrast, the hAlu genes were not detected in the brain, mandibular lymph nodes, pancreas, mesenteric lymph nodes, bone marrow, or tail of all mice. At the 1-day time point, the hAlu genes were detected only in female spleens (female: 5 out of 5) and some of the female kidneys (female: 3 out of 5), and all of the heart, lungs, liver, and blood of both male and female mice. At the 3-day time point, the hAlu genes were detected in the heart and lungs of all mice, and only in the blood of 2 out of 5 female mice. At the 1-week time point, the hAlu genes were detected in the heart and lungs of all mice, and at the 2-week time point, the hAlu genes were only detected in some lungs (males: 4 out of 5, females: 3 out of 5). At the 4-, 6-, and 8-week time points, the hAlu genes were not detected in all mice (Fig. 5A).

The distribution results revealed that the single-administered UDSCs remain in the blood and circulate for up to 3 days after administration. The administered UDSCs were cleared out from most organs, excluding the lungs, by the end of the first week, and the residual UDSCs in the lungs were completely cleared by the fourth week. There was no significant difference in the organ-specific retention period of UDSCs between males and females.

### Biodistribution of UDSCs following repeated administration

The distribution patterns in major organs were evaluated at 2 h, 1 day, 3 days, 1 week, 2 weeks, 4 weeks, 6 weeks, and 8 weeks after repeated intravenous administration (triple injections, 1-week intervals) of UDSCs into BALB/c nude mice.

At the 2-h time point, in a subset of mice, the hAlu genes were detected in the brain (females: 2 out of 5), and kidneys (males: 1 out of 5, females: 2 out of 5) as well as in all of the heart, lungs, liver, spleen, and blood of both male and female mice. At the 1-day time point, the hAlu genes were detected in the lungs of all mice. Furthermore, in a subset of mice, the hAlu genes were detected in the heart (males: 2 out of 5, females: 2 out of 5), and liver (females: 1 out of 5). At the 2-week time point, the hAlu genes were only detected in some lungs (males: 2 out of 5, females: 2 out of 5). At the 5-week time point, the hAlu genes were not detected in all mice (Fig. 5B).

The distribution results revealed that the triple-administered UDSCs remained only in the lungs of some mice from 2 weeks after the last administration. The residual hAlu genes in the lungs gradually decreased to levels of LLOQ (10 pg) by the fourth week (1 out of 5 males: 13.28 pg, 1 out of 5 females: 19.25 pg). In conclusion, under the conditions of this experiment, there was no significant difference in the retention period following both single and repeated administration of UDSCs.

### Long-term tumorigenicity of UDSCs following repeated administration

The potential of tumorigenicity was examined over a period of 26 weeks following the repeated intravenous and subcutaneous administration of UDSCs (quadruple injection, 1-week intervals) into BALB/c nude mice.

**Fig. 6:**
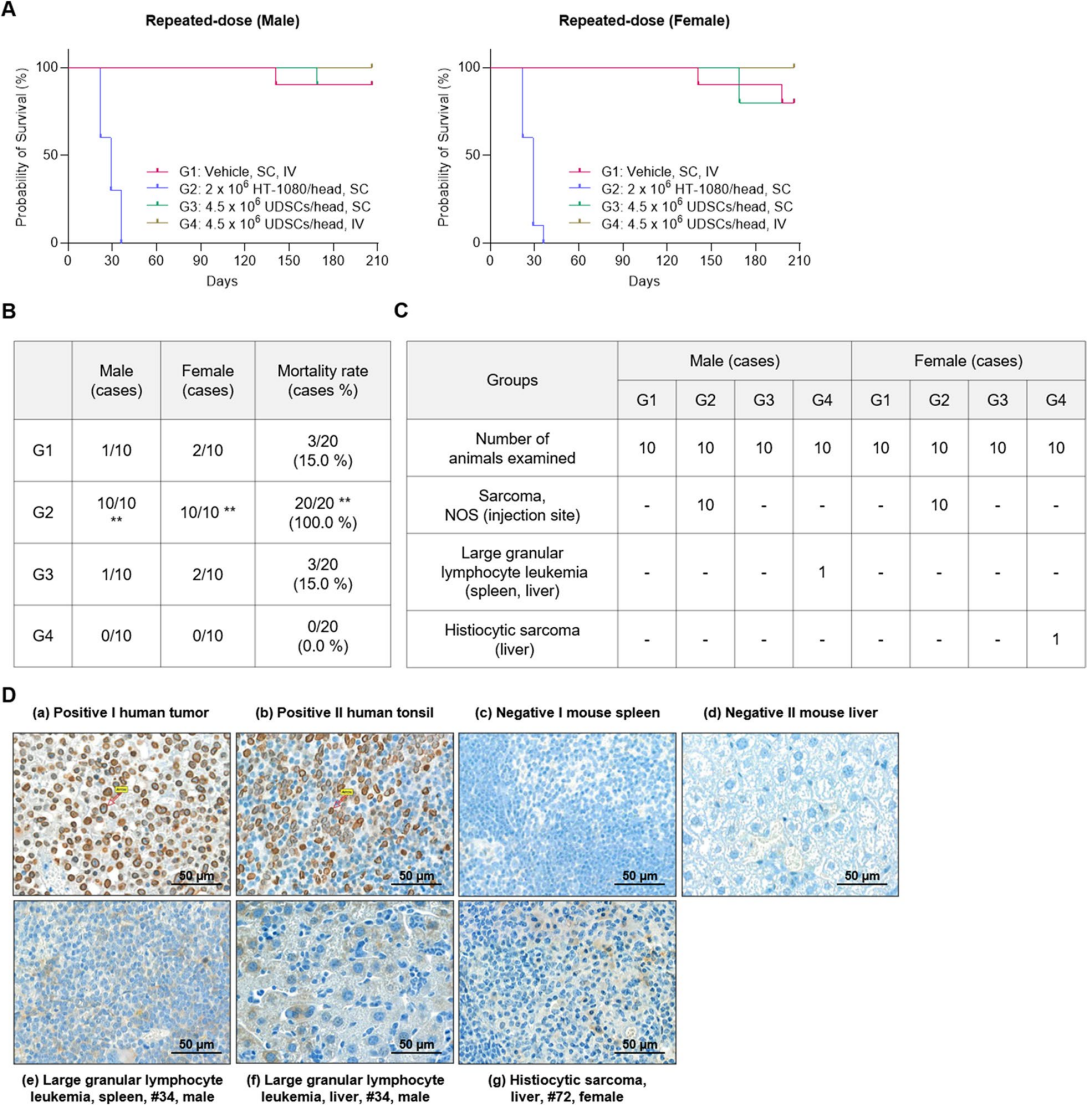
Long-term monitoring of tumorigenicity following repeated administration of UDSCs. **A** Tumorigenic potential was examined during 26 weeks after repeated subcutaneous and intravenous administration of UDSCs. **B** UDSCs-administered groups (G3, G4) exhibited no significant changes in mortality rate compared to the vehicle control group (G1). **C** Tumor formation was assessed in all groups during the final necropsy. **D** Immunohistochemistry analysis on tumor tissues from the intravenous administration group (G4) was conducted to determine the origin of tumors. **p < 0.01. NOS, not otherwise specified

In the positive control group (G2), significant nodule enlargement and ulcerations were observed at the injection sites in both male and female mice. Based on the assessment that the mice experienced severe distress, the decision was made to euthanize them on day 23–30 of the examination to alleviate their suffering (Fig. 6A). During the examination period, premature death or natural death in the UDSCs-administered group (G3, G4) did not show a significant difference in mortality rate compared to the vehicle control group (G1) (Fig. 6B). Since there were no consistent observed changes that may cause death, the variation in mortality rate is determined to be the natural cause rather than the influence of UDSCs administration.

In the UDSCs-administered group (G3, G4), general symptoms such as emaciation, erosion, scratched wounds, crust formation, decreased spontaneous movement, edematous, exfoliation, abdominal distention, prolapse of the penis, rectal prolapse, soft stool, bent tail, and necrosis were observed. However, these symptoms without accompanying weight changes were observed in the vehicle control group (G1) as well. These changes were either transient or infrequent occurrences. Therefore, it was determined that such variations in general symptoms were unrelated to the administration of UDSCs (data were not shown).

Each group underwent a necropsy to observe the presence of tumor formation and any macroscopic abnormalities were further examined by histopathological analysis. In the positive control group (G2), tumor formation was observed at the injection sites in all mice, and these were identified as mesenchyme origin unspecified malignant tumors (sarcomas). Any notable histological changes were not observed in UDSCs subcutaneous administration group (G3) except for spontaneous findings that can also occur in BALB/c nude mice of the same age. In the UDSCs intravenous administration group (G4), significant abnormal proliferation and tumor formation were observed in one female and one male mouse each, compared to the vehicle control group (G1). Histopathological analysis revealed that large granular lymphocyte leukemia occurred in the spleen and liver of one male mouse (#34), and histiocytic sarcoma occurred in the liver of one female mouse (#72) in group G4 (Fig. 6C).

To determine whether the tumors were induced by the administration of UDSCs, the origin of tumors was evaluated by immunohistochemistry analysis using human-specific anti-Lamin A and anti-Lamin C antibodies. In the immunohistochemistry results, the positive staining control tissues exhibited a brown ring on the nuclear membrane of the human tumor cells. However, negative staining control tissues and tumor tissues from #34 and #72 mice were negative for staining (Fig. 6D). It was conclusively determined that the tumor formations observed in the G4 group are not of human origin, indicating that the tumors are unrelated to UDSCs. It also has been reported that spontaneous tumor formations were commonly observed in tumorigenicity or long-term toxicity studies using BALB/c nude mice.

## Discussion

Cell therapeutics is a pharmaceutical manufactured by isolating and culturing specific autologous, allogeneic, or xenogeneic cells, or modifying the biological characteristics of those cells [34]. It is used for the purpose of restoring the function of damaged tissues and organs. Various types of cells can be used for cell therapeutics. Among them, stem cells are the most extensively researched as an alternative treatment method for incurable diseases. The distinctive features of stem cells, such as self-renewal, differentiation potential, and homing effect, set them apart from common somatic cells [35]. However, national regulatory agencies such as the U.S. Food and Drug Administration (FDA) and the European Medicines Agency (EMA) recommend comprehensive consideration of the safety of stem cells through preclinical trials before the clinical application because of its distinctive features [36, 37]. Therefore, in this study, we aimed to investigate the biological characteristics, safety, and in vivo distribution profile of GMP-manufactured UDSCs.

First, we developed a culture method for UDSCs that enhances the expression of Klotho, a potential renal therapeutic factor. And we optimized the GMP manufacturing systems for the isolation and large-scale expansion of Klotho-enhanced UDSCs. UDSCs demonstrated multipotency by differentiating into adipocytes, osteocytes, and chondrocytes. They also expressed MSCs stemness markers CD73 and CD105, as well as specific UDSCs-positive markers CD146 and SSEA-4. Furthermore, under the established culture conditions, the expression of Klotho in UDSCs was significantly enhanced and the biological characteristics of UDSCs remained consistent and reproducible. To assess their therapeutic potential, we examined the homing capability of Klotho-enhanced UDSCs to kidneys damaged by ischemia–reperfusion. Our data revealed that UDSCs effectively migrated to the injured renal tissues. Importantly, the reduced renal Klotho expression observed in the IRI group was recovered following the administration of UDSCs. This underscores the therapeutic potential of Klotho-enhanced UDSCs in the treatment of kidney diseases.

Next, the non-clinical toxicity test was conducted to identify potential toxicity and explore safe dosages and administration routes for the clinical application of UDSCs. To evaluate the toxicity of UDSCs, we conducted tests using BALB/c nude mice, a widely used immunodeficient mouse model. The results revealed that the ALD of UDSCs following a single intravenous administration was above 4.5 × 10^6^ cells/head (high-dose). Furthermore, when high-dose of UDSCs were repeatedly administered intravenously (quadruple injection, 1-week intervals), no toxicological or immunological adverse effects were observed.

Moreover, the qPCR analysis was performed using a primer and probe set specific to the hAlu gene to assess the distribution pattern and retention period of UDSCs in different organs. The analysis allowed for specific and quantitative evaluation of the presence and duration of UDSCs in various organs. The results revealed that the administered UDSCs persisted in the blood and circulated, distributed into major organs such as the heart, lungs, liver, pancreas, and kidneys for up to 3 days after the last administration. Additionally, it was determined that UDSCs remaining in major organs were cleared within 2 weeks, with the exception of the lungs. UDSCs that remained in the lungs also gradually declined and were completely cleared within 4–5 weeks. In conclusion, our results demonstrate that the administration frequency of UDSCs, single and repeated administrations (triple injection, 1-week intervals), did not exert a significant impact on their distribution and residence period. Moreover, the results indicate that UDSCs did not exhibit targeted distribution or retention in specific organs of both normal male and female mice.

These data on toxicity and distribution according to the administration dosage, frequency, and route can provide in vivo safety information for the clinical application of UDSCs. However, there are challenges in applying typical pharmacokinetics (PK) to cell therapeutics, and extrapolating animal dosage to clinical trials can be limited due to species specificity and immunogenicity issues. Therefore, in the final therapeutic strategy and clinical application of cell therapeutics, a comprehensive review of all conducted examinations' results should be undertaken to make informed decisions.

Lastly, due to the unique characteristics of stem cells, long-term monitoring of tumorigenic potential is one of the most crucial aspects to consider for the safety of stem cell therapeutics. The risk of tumorigenesis can vary depending on factors such as cell origin, genetic modification, differentiation potential, and administration route. To assess the tumorigenicity of UDSCs, we conducted a study where UDSCs were repeatedly administered to BALB/c nude mice through subcutaneous and intravenous routes (quadruple injection, 1-week intervals), and tumor occurrence was monitored for 26 weeks. In conclusion, there was no direct correlation found between UDSCs administration and the histopathological findings in deceased mice from subcutaneous UDSCs administration. Additionally, the tumors observed in a few mice after intravenous UDSCs administration were confirmed to be of non-human origin, ultimately determining that UDSCs do not possess tumorigenic potential.

As CKD progresses, serum Klotho levels decrease in patients. Recent studies have shown a strong correlation between reduced cellular Klotho expression and the onset of CKD. Several studies have been conducted to treat CKD using recombinant Klotho protein [38–40]. However, there is a limitation in its therapeutic efficacy because Klotho protein cannot exclusively target damaged renal tissues. Based on these studies, we have developed a CKD treatment strategy using patient-specific UDSCs with enhanced Klotho expression, rather than simply cultured UDSCs. Our results demonstrate that Klotho-enhanced UDSCs possess excellent homing ability to damaged kidneys and can enhance the expression levels of Klotho in renal tissues.

Despite the advantages for clinical application, such as non-invasive collection, large-scale expandability, and therapeutic potential, non-clinical safety and in vivo distribution studies of UDSCs have not yet been reported. In this study, we demonstrated that Klotho-enhanced UDSCs can be successfully manufactured as clinical cell therapeutics under GMP regulations. Additionally, the endogenous Klotho enhancement using a GMP-compliant custom medium did not induce significant toxicological changes following administration in animal models. Furthermore, we confirmed that Klotho enhancement did not lead to abnormal distribution or retention in major organs.

## Conclusions

Our systemic evaluation results of non-clinical safety and biodistribution demonstrate for the first time that Klotho-enhanced UDSCs manufactured in GMP facility can be safely administered intravenously without inducing toxicity or tumorigenicity over an extended period. Based on the overall results, we propose the clinical applicability of UDSCs.

### Supplementary Information

**Additional file 1. Table S1:** Antibodies for flow cytometry, western blotting, and immunofluorescence analysis.

**Additional file 2. Fig. S1:** Full-length original blot images of Figure 1C.

## Data Availability

The data presented in this study are available on request from the corresponding author.

## Abbreviations

AKI: Acute kidney injury
ALB: Albumin
ALD: Approximate lethal dose
ALP: Alanine aminotransferase
AR: Analytical recovery
AST: Aspartate aminotransferase
BUN: Blood urea nitrogen
BW: Body weight
CD: Cluster of differentiation
CKD: Chronic kidney disease
CPK: Creatinine phosphokinase
CRE: Creatinine
EMA: European Medicines Agency
EMT: Epithelial–mesenchymal transition
EOS: Eosinophils
FDA: Food and Drug Administration
FSC: Forward scatter
GLP: Good laboratory practice
GLU: Glucose
GMP: Good manufacturing practice
HGB: Hemoglobin
HT-108: Human fibrosarcoma cell line
INHAND: International Harmonization of Nomenclature and Diagnostic Criteria
IRB: Institutional review board
IV: Intravenous
KSFM: Keratinocyte serum-free medium
LLOQ: Lower limit of quantification
LN: Lymph node
LOD: Limit of detection
LYM: Lymphocytes
MONO: Monocytes
MPV: Mean platelet volume
MSCs: Mesenchymal stem cells
NBF: Neutral buffered formalin
NEU: Neutrophils
NGAL: Neutrophil gelatinase associated lipocalin
NOS: Not otherwise specified
NTC: No template control
PK: Pharmacokinetics
PLT: Platelet count
PSCs: Pluripotent stem cells
RBC: Red blood cell
RSD: Relative standard deviation
SC: Subcutaneous
SOP: Standard operating procedure
SSC: Side scatter
SSNDC: Standardized System of Nomenclature and Diagnostic Criteria
STP: American Society of Toxicologic Pathology
TCHO: Total cholesterol
TG: Triglyceride
TP: Total protein
UDSCs: Urine-derived stem cells
WBC: White blood cell
