# Supplementary material for: In vivo safety and biodistribution profile of Klotho-enhanced human urine-derived stem cells for clinical application
Source: Stem Cell Res Ther. 2023 Dec 10;14:355. doi: 10.1186/s13287-023-03595-y (PMC10712141; doi:10.1186/s13287-023-03595-y)
Supplement: Supplementary file 1 — Additional file 1. Table S1: Antibodies for flow cytometry, western blotting, and immunofluorescence analysis. [file 13287_2023_3595_MOESM1_ESM.docx]

**Additional file 1**

**Table S1.** Antibodies for flow cytometry, western blotting, and immunofluorescence analysis

| Analysis | Antibody | Manufacturer | Catalog no. |
| --- | --- | --- | --- |
| Flow cytometry | FITC Anti-human CD73 | BD Biosciences | 561254 |
|  | PE Anti-human CD90 |  | 555596 |
|  | FITC Anti-human SSEA4 |  | 560126 |
|  | PE Anti-human CD146 |  | 550315 |
|  | FITC Mouse Anti-human CD31 |  | 555445 |
|  | PE Anti-human CD34 |  | 555822 |
|  | FITC Anti-human CD45 |  | 555482 |
| Western blotting | Anti-Klotho mAb (Clone KM2076) | Cosmo bio | KO603 |
|  | Anti-β-Actin Antibody (C4) | Santacruz | sc47778 |
| Immunofluorescence | Anti-Klotho (F-5) | Santacruz | sc515939 |
|  | Goat anti-Mouse IgG (H+L) Cross-Adsorbed Secondary Antibody, Alexa Fluor 488 (green) | Invitrogen | A-11001 |
