# Supplementary material for: In vivo safety and biodistribution profile of Klotho-enhanced human urine-derived stem cells for clinical application
Source: Stem Cell Res Ther. 2023 Dec 10;14:355. doi: 10.1186/s13287-023-03595-y (PMC10712141; doi:10.1186/s13287-023-03595-y)

Additional file 2

Fig. S1. Full-length original blot images of Figure 1C (upper: Klotho / lower: β-actin).

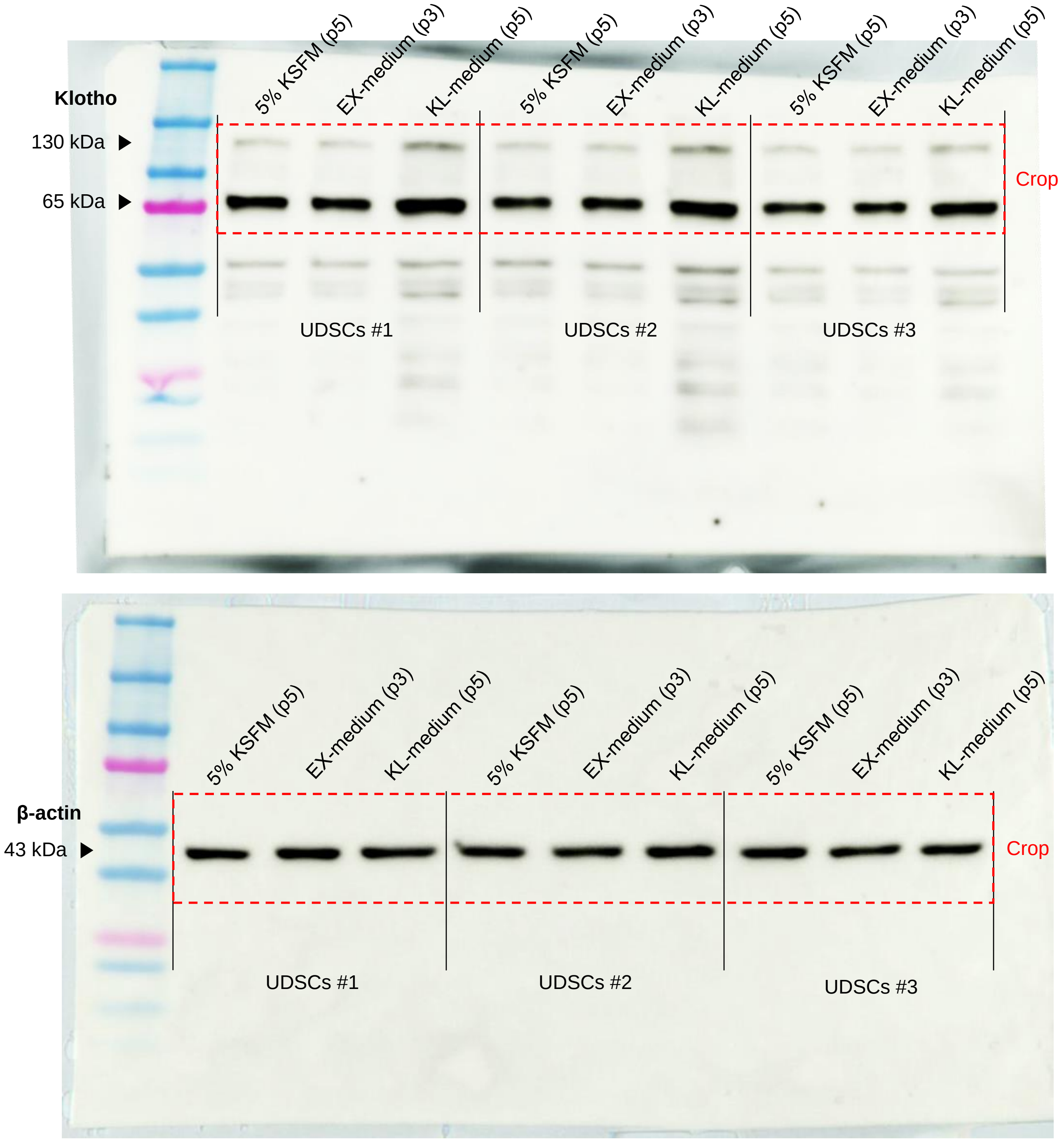

Supplement: Supplementary file 2 — Additional file 2. Fig. S1: Full-length original blot images of Figure 1C. [file 13287_2023_3595_MOESM2_ESM.pdf]
